# Supplementary material for: Base-Free Oxidation of HMF to FDCA over Ru/Cu-Co-O·MgO under Aqueous Conditions
Source: Molecules. 2024 Jul 6;29(13):3213. doi: 10.3390/molecules29133213 (PMC11243489; doi:10.3390/molecules29133213)
Supplement: Supplementary file 1 [file molecules-29-03213-s001.zip › molecules-3044476-supplementary.pdf]

Supporting Information

For

Base-Free Oxidation of HMF to FDCA over Ru/Cu-Co-O·MgO under Aqueous Conditions

Experimental section

Chemicals and materials

Copper nitrate trihydrate ($Cu(NO_3)_2 \cdot 3H_2O$, 99%), Cobalt nitrate hexahydrate ($Co(NO_3)_2 \cdot 6H_2O$, 99%), Magnesium acetate tetrahydrate ($Mg(CH_3COO)_2 \cdot 4H_2O$, 99%), sodium borohydride ($NaBH_4$, 97%), were all purchased from Sinopharm Chemical Reagent Co., Ltd. Sodium hydroxide (NaOH, 96%) was acquired from Tianjin Damao Chemical Reagent Factory. 5-Hydroxymethylfurfural (HMF, 99%), 2,5-furandicarboxylic acid (FDCA, 97%), and 5-hydroxymethyl-2-furancarboxylic acid (HFCA, 98%) were all obtained from Shanghai Debo Biochemical Technology Co., Ltd. 5-Formyl-2-furancarboxylic acid (FFCA, 98%), methanol (CH_3OH, chromatographic grade), formic acid (HCOOH, chromatographic grade) were acquired from Shanghai Aladdin Biochemical Technology Co., Ltd. Trichlorotrihydrate ruthenium ($RuCl_3 \cdot 3H_2O$, 99%) were purchased from Tianjin Baima Technology Co., Ltd. Except for magnesium acetate tetrahydrate, which required heating to a constant weight at 140 ℃ to obtain anhydrous magnesium acetate, all chemicals were used directly without further purification.

Catalyst characterizations

Powder X-ray diffraction (XRD) tests were conducted using Bruker D8 Advance

instrument with Cu-Kα radiation, a tube voltage of 40 kV, a scan range of 5-80°, a scan step of 4° per min. N_2 physical adsorption-desorption experiments were carried out on a Micromeritics TriStar II Plus 2.02 instrument. Before testing, samples were degassed under N_2 atmosphere at 120 °C for 12 h, followed by adsorption-desorption at liquid nitrogen temperature (-196 °C). X-ray photoelectron spectroscopy (XPS) analyses were performed using an Escalab 250Xi instrument with AlKα radiation. The binding energies of all elements were referenced to C1s, with energy calibration done at 284.8 eV. Energy dispersive spectroscopy (EDS) analysis was carried out with a Bruker XFlash detector on a scanning electron microscope (SEM, JEOL-JSM-7610F), with a working voltage of 15 kV, to determine the elemental composition and distribution on the sample surface. Transmission electron microscopy (TEM) characterizations were conducted using Talos F200X equipment to observe the microstructure and lattice fringes of the samples. Samples were dispersed in anhydrous ethanol before testing. Inductively coupled plasma optical emission spectroscopy (ICP-OES) analyses were performed using an Agilent 720 instrument to determine the elemental content in the post-reaction solutions. CO_2 temperature-programmed desorption (CO_2-TPD) tests were performed using an AutoChem II 2920 instrument. Prior to characterization, samples were pretreated in He (20 mL/min) at 200 °C for 1 h, then cooled to 50 °C to adsorb CO_2 for 30 min (20 mL/min), followed by purging with He (20 mL/min) at 80 °C for 30 min to remove physically adsorbed CO_2. The CO_2-TPD data of the samples were recorded by increasing the temperature from 100 °C to 800 °C at a rate of 10 °C/min. H_2 temperature-programmed reduction (H_2-TPR) tests were conducted using an

AutoChem II 2920 instrument. Before characterization, the sample was placed in a quartz tube fixed with quartz wool and pretreated in He atmosphere (20 mL/min) at 180 °C for 1 h. Then, the sample was cooled to 50 °C, and the H_2-TPR measurement was performed using 5% H_2 and 95% Ar reducing gas mixture at a flow rate of 20 mL/min, with a temperature ramp of 10 °C/min up to 800 °C. Nuclear Magnetic Resonance spectroscopy (NMR) analysis was performed using an Advance III 400 MHz nuclear magnetic resonance spectrometer. During the test, DMSO was used as the solvent, and FDCA was characterized accordingly.

Product and reaction analysis

The detection wavelengths for HFCA, FDCA, HMF, FFCA and DFF were 250, 264, 284, 287, and 288 nm, respectively.

The conversion ratio of HMF (mol%) can be calculated using the formula (1).

$$\text{The conversion of HMF (mol\%)} = \frac{\text{Moles (HMF converted)}}{\text{Moles (initial HMF)}} \times 100\% \qquad (1)$$

The yield of FDCA (mol%) can be calculated using the formula (2).

$$\text{The yield of FDCA (mol\%)} = \frac{\text{Moles (FDCA produced)}}{\text{Moles (initial HMF)}} \times 100\% \qquad (2)$$

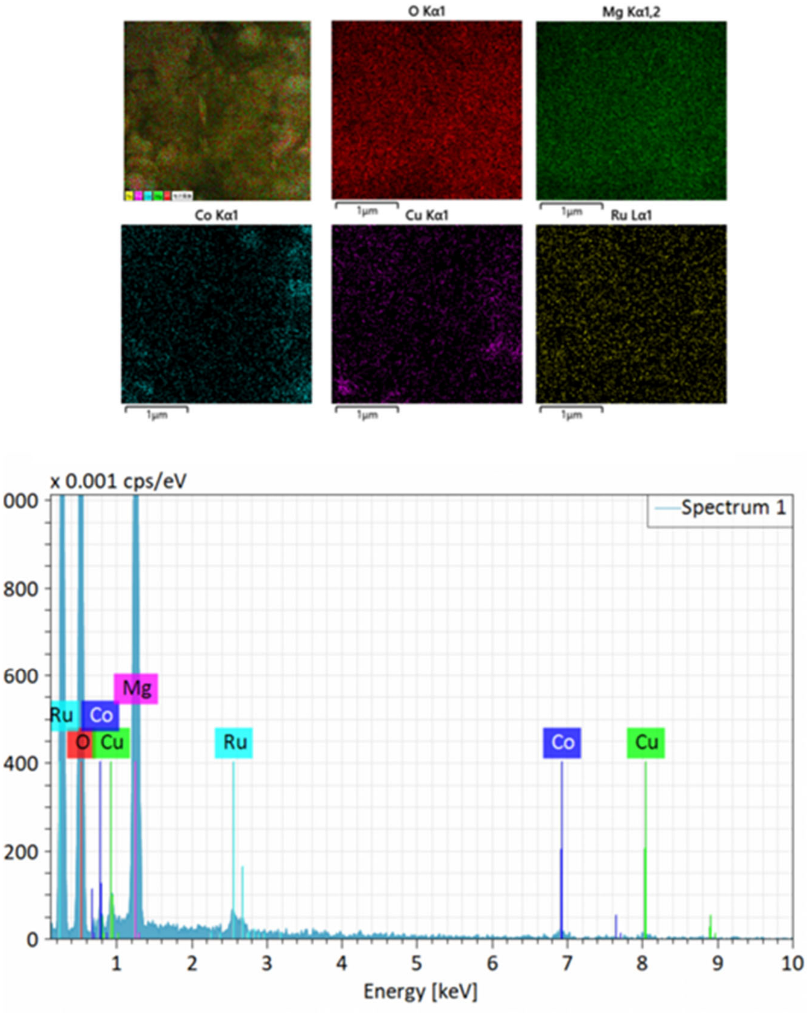

Figure S1. EDS Spectrum and element mapping patterns of Ru_4/Cu1-Co1-O·MgO.

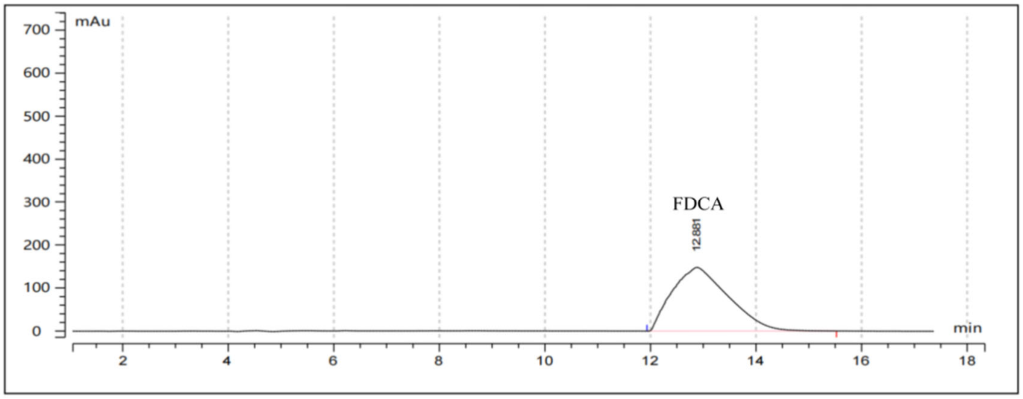

Figure S2. Semi-preparative liquid chromatogram.

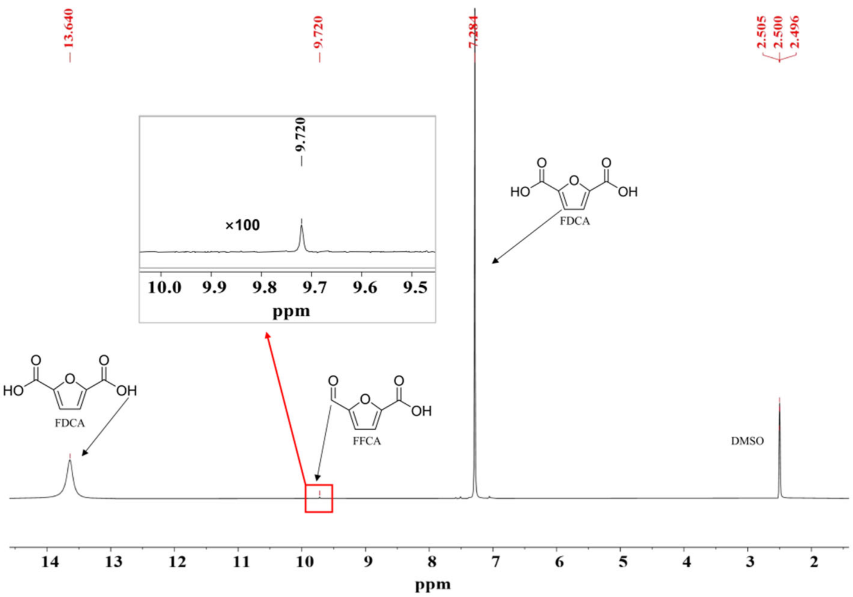

Figure S3. The ^{1}H-NMR spectrum of the separated FDCA obtained.
